# The Effect of Bio-Synthesized Silver Nanoparticles on Germination, Early Seedling Development, and Metabolome of Wheat (*Triticum aestivum* L.)

**DOI:** 10.3390/molecules27072303

**Published:** 2022-04-01

**Authors:** Lesław Bernard Lahuta, Joanna Szablińska-Piernik, Katarzyna Głowacka, Karolina Stałanowska, Viorica Railean-Plugaru, Marcin Horbowicz, Paweł Pomastowski, Bogusław Buszewski

**Affiliations:** 1 Department of Plant Physiology, Genetics and Biotechnology, University of Warmia and Mazury, Oczapowskiego Street 1A/103, 10-719 Olsztyn, Poland; joanna.szablinska@uwm.edu.pl (J.S.-P.); kasiag@uwm.edu.pl (K.G.); karolina.stalanowska@uwm.edu.pl (K.S.); marcin.horbowicz@uwm.edu.pl (M.H.); 2 Interdisciplinary Center for Modern Technologies, Nicolaus Copernicus University in Torun, 87-100 Torun, Poland; rviorela@yahoo.com (V.R.-P.); pawel_pomastowski@wp.pl (P.P.); bbusz@chem.umk.pl (B.B.); 3 Department of Environmental Chemistry and Bioanalytics, Faculty of Chemistry, Nicolaus Copernicus University in Torun, 87-100 Torun, Poland

**Keywords:** wheat, seedling, silver nanoparticles, ROS, metabolic profiles

## Abstract

Changes in the metabolome of germinating seeds and seedlings caused by metal nanoparticles are poorly understood. In the present study, the effects of bio-synthesized silver nanoparticles ((Bio)Ag NPs) on grains germination, early seedlings development, and metabolic profiles of roots, coleoptile, and endosperm of wheat were analyzed. Grains germinated well in (Bio)Ag NPs suspensions at the concentration in the range 10–40 mg/L. However, the growth of coleoptile was inhibited by 25%, regardless of (Bio)Ag NPs concentration tested, whereas the growth of roots gradually slowed down along with the increasing concentration of (Bio)Ag NPs. The deleterious effect of Ag NPs on roots was manifested by their shortening, thickening, browning of roots tips, epidermal cell death, progression from apical meristem up to root hairs zone, and the inhibition of root hair development. (Bio)Ag NPs stimulated ROS production in roots and affected the metabolic profiles of all tissues. Roots accumulated sucrose, maltose, 1-kestose, phosphoric acid, and some amino acids (i.e., proline, aspartate/asparagine, hydroxyproline, and branched-chain amino acids). In coleoptile and endosperm, contrary to roots, the concentration of most metabolites decreased. Moreover, coleoptile accumulated galactose. Changes in the concentration of polar metabolites in seedlings revealed the affection of primary metabolism, disturbances in the mobilization of storage materials, and a translocation of sugars and amino acids from the endosperm to growing seedlings.

## 1. Introduction

The growth of the world population increases the demand for food and especially the yield of major crops such as maize, rice, and wheat. Wheat and its products provide about 21% of the world’s food energy and protein needs [1]. Therefore, it is important to increase wheat yield by coping with adverse environmental factors caused by climate change, soil degradation, and insect and disease hazards. To meet these challenges, it is necessary to introduce innovative technologies into modern agriculture and among them includes nanotechnology [2,3,4].

Nanotechnology deals with the production and study of the properties of fine particles between 1 and 100 nm in size called nanoparticles (NPs). NPs are intentionally designed and produced for specific properties related to their size and shape, as well as surface and chemical features. Phytonanotechnology, i.e., the application of NPs in plant science and crop production systems, is currently of growing interest [3,5]. NPs are used in agriculture as nanofertilizers, nanopesticides, nanoherbicides [5], nanosensors, and agrochemical-encapsulated nanocarrier systems [4]. There are known four main types of nanoparticles (NPs): carbon, ceramic, metallic, and polymeric [2]. Metal oxide NPs and metal NPs, due to their antimicrobial properties, have the ability to control and combat both plant pests and phytopathogens. Therefore, the application of nanoparticles of zinc oxide (ZnO NPs), titanium dioxide (TiO_2_ NPs), copper (Cu NPs), and silver (Ag NPs) for plant disease management is currently a major part of phytonanotechnology in agriculture [5,6,7,8,9,10]. However, the use of NPs as fungicides, bactericides, and insecticides can also affect plants [11] and can contaminate the environment, agricultural products, and food itself [12]. Due to their small size and high surface activity, metallic NPs are mostly harmful to organisms as they readily cross biological membranes, exhibiting immediate or delayed toxic effects [12,13]. The effects of NPs on plants depend both on their chemical nature, size, shape, coating agents, and concentration, as well as on the plant species and its developmental stage [8,9,10,11,12,14,15,16,17]. Therefore, the safety of the use of metal NPs in crops requires detailed studies.

The results of recently published papers indicate that the practical use of NPs in wheat cultivation is still in the early stages of application. Some of the metal oxide NPs such as TiO_2_ NPs and ZnO NPs, as well as Ag NPs, have been shown to enhance seed germination, plant growth, and the yield of wheat, but when applied at strictly selected concentrations [1,17]. Moreover, they can alleviate crop stress conditions such as drought, salinity, and heat [5], and they can control wheat pathogens [6,7,18,19] due to their antifungal, antiviral, and bactericidal properties [6,20]. For example, Ag NPs applied at the appropriate concentration can be equally or more effective compared to synthetic fungicides [21] or antibiotics [22]. However, too high a dose of Ag NPs is phytotoxic for monocots and dicots model plants [17] as well as crops [8]. Silver NPs show multiple modes of inhibitory action against microorganisms [23]. The mechanism of toxicity of Ag-NPs and silver ions mainly involves cellular dysfunction caused by cell membrane damage. Additionally, the structures of intracellular molecules (proteins, lipids, and DNA) can be damaged and oxidative stress can be induced through the synthesis of reactive oxygen species (ROS) and the accumulation of free radicals [6,20,24].

The effects of Ag NPs on plants depend on their characteristics related to the method of synthesis and the pelleting agents used and the concentration applied [25,26]. Therefore, the effects of Ag NPs on wheat productivity can vary and result in plant growth inhibition, delayed grain maturity, and reduced yield quality [24,27,28,29]. It should be noted that such effects were observed during a four-month application of Ag NPs at a concentration of 2 g/kg soil, which is relatively high. However, several-fold lower concentrations had not affected the rate of seed germination, plant growth, and amino acids composition in grains [30].

The majority of studies conducted to date on the phytotoxicity of Ag NPs have been concerned with short-term exposure to it and the effects on essential physiological responses such as seed germination, root growth, and nanoparticle transport and accumulation. The results of these studies showed that in germinating grains and young wheat seedlings treated with Ag NPs, there were large disturbances in root morphology, a partial disintegration of cell membranes, and a peroxidation of lipids contained therein accompanied by oxidative stress and a decrease in chlorophyll, carotenoids, and protein content [24,31,32,33,34]. Moreover, there was an accumulation of some protective compounds such as proline [34]. Similar effects of Ag NPs were found in germinating seeds and seedlings of other plants [35,36]. However, detailed changes in the metabolome of wheat seedlings treated with Ag NPs are not yet known. Moreover, each new method of NPs production means that the properties of the product are not fully known [20,26,28], and without conducting investigations, it is not possible to decide on their effect on the plant.

This paper presents the results of a study including detailed biometric, microscopic, and metabolic measurements of the effect of Ag NPs obtained using *Lactobacillus paracasei* on germination, seedlings development, and changes in the metabolic profiles of roots, coleoptile, and endosperm of three-day-old seedlings of wheat. The effect of Ag NPs applied at concentrations disturbing seedling development changes the content of primary metabolites: carbohydrates, amino acids, and organic acids in young seedlings were revealed.

## 2. Results and discussion

### 2.1. Properties of Bio-Synthesized Ag NPs

**Figure 1:**
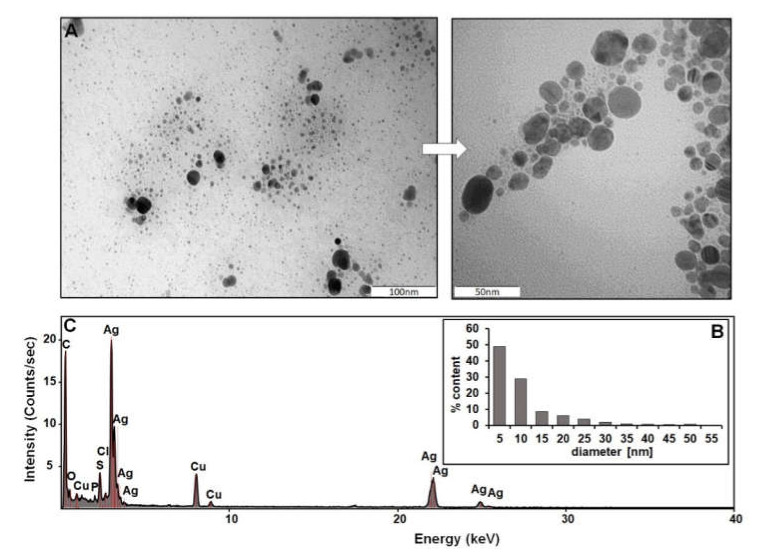
Characteristics of (Bio)Ag NPs: TEM image of Ag NPs (**A**), size distribution (**B**), energy-dispersive X-ray spectroscopy (EDS), and analysis (**C**).

The size and morphology of biologically synthesized Ag NPs are shown in Figure 1A. For Ag NPs of varying sizes, most of the nanoparticles were found to be spherical and monodispersed. Figure 1B provides details on particle size range determined using TEM analysis; the diameters of more than 75% of the counted Ag NPs ranged between 5 and 10 nm and ca 20% between 15 and 25 nm. At least 483 nanoparticles have been assessed and represented in the form of a histogram in order to determine the predominant population. Considering the fact that biologically synthesized NPs comprise a metallic core and organic deposit, the stability of the system will depend on both the silver metallic core and the organic coating, as was previously described by Railean et al. (2020) [37]. Such a nanocomposite synthesized by a suitable bacterial strain was stable for at least 6 days. Since the details of the characterization of NPs have already been published, only their composition and metallic form are highlighted in this work to describe the complexity of the biocolloid structure.

Additionally, energy-dispersive X-ray spectroscopy confirmed the presence of both metallic silver registered at 2.7–3.2 keV as well as the presence of other elements (carbon, oxygen, phosphor, and sulfur); organic components recorded in (Bio)Ag NPs sample are related to the organic part naturally coating the metallic silver core during the synthesis process (Figure 1C). The additional recorded signals of copper elements come from TEM grinding used for the analysis.

### 2.2. Preliminary Experiment on Phytotoxicity of Ag NPs

Ag NPs at 20 and 40 mg/L did not affect the germinability and percentage of developing seedlings of the three wheat cultivars evaluated (Table 1). However, the investigated nanoparticles applied at concentration 20 mg/L clearly inhibited the growth of both primary roots and the coleoptile.

When twice the concentration of (Bio)Ag NPs (40 mg/L) was applied, the effect on coleoptile was similar to that at 20 mg/L, but there was a strong inhibition of growth and development of the root system, regardless of the wheat cultivar tested (Supplementary Materials, Figure S1). This was accompanied by a decrease in the fresh biomass of seedlings in all wheat cultivars. However, a marked reduction in seedling dry weight growth was observed only in the cultivar ‘Ostka Strzelecka’ (Table 1). Therefore, this wheat cultivar was selected for a further phytotoxicity study of (Bio)Ag NPs.

### 2.3. Phytotoxic Effects of Ag NPs on Wheat Seedlings

**Figure 2:**
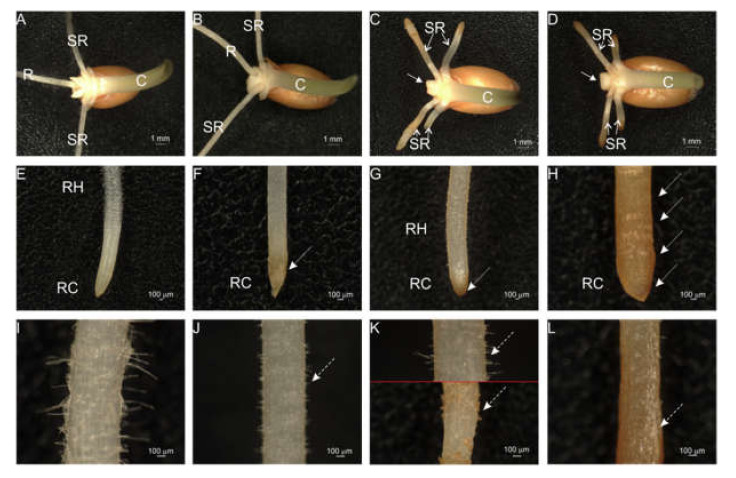
The morphology of 3-days-old seedlings of wheat (*Triticum aestivum* L., cv. ‘Ostka Strzelecka’) developing in the water (**A**,**E**,**I**) or (Bio)Ag NPs at 10 (**B**,**F**,**J**), 20 (**C**,**G**,**K**) or 40 mg/L (**D**,**H**,**L**). Abbreviation: C—coleoptile; R—primary root; RC—root cap; RH—root hairs zone; SR—seminal roots. The arrows indicate morphological disturbances caused by (Bio)Ag NPs.

Wheat grains of the cultivar ‘Ostka Strzelecka’ showed a high germinability (93–97%) independent of the (Bio)Ag NPs concentration applied. This observation is consistent with the results of previous studies, which showed no effects on Ag NPs with concentrations up to 75 mg/L (with sizes of 10–35 nm and having different coating agents) on wheat germination [25,28,32,37,38]. However, the initial growth of wheat seedlings was strongly affected by (Bio)Ag NPs at concentrations of 20 and 40 mg/L. Primary root elongation decreased with increasing (Bio)Ag NPs concentration, while coleoptile growth was slowed by about 25%, regardless of the used nanoparticles concentration. (Bio)Ag NPs doses of 20 and 40 mg/L inhibited the elongation of the first and second seed–root pairs. In addition, the frequent inhibition of primary root development was observed (Figure 2C,D).

**Figure 3:**
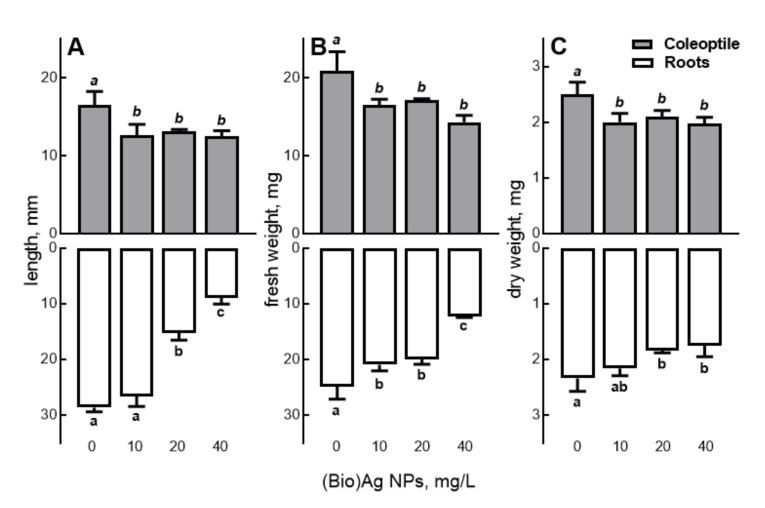
The effect of (Bio)Ag NPs on the length of primary seminal root and coleoptile (**A**), fresh weight (FW, **B**), and dry weight (DW, **C**) of roots and coleoptile of a 3-days-old seedling of wheat (*Triticum aestivum* L., cv. ‘Ostka Strzelecka’). Control grains germinated in double-distilled water. Values are means (*n* = 3) ± SD. Bars with the same letters (a–c, *a–b*) are not significantly (*p* < 0.05) different after the ANOVA test and Tukey’s post hoc corrections. On graph A, the length of the primary seminal root is shown.

Wheat seedlings treated with (Bio)Ag NPs at 10 and 20 mg/L resulted in the development of brown coloring of the root caps (Figure 2F,G, dashed line arrows). In seedlings grown in the presence of (Bio)Ag NPs at 40 mg/L, root tips were swollen and brown, and browning spread from the meristematic zone to the elongation zone (Figure 2H, dashed arrows). Moreover, in seedlings treated with (Bio)Ag NPs in a concentration-dependent manner, there was a disturbance in the growth of root hairs that were significantly shorter than in the control (Figure 3G–L). The root morphology abnormalities found in this study are similar to those shown in seedlings of barley [38,39], switchgrass [39,40], and *Arabidopsis thaliana* [40,41], and this confirms the dose-dependent phytotoxicity of Ag NPs (Table 1).

After the application of (Bio)Ag NPs at 20 mg/L, the primary root was two-times shorter and as much as three-times shorter when (Bio)Ag NPs were used at 40 mg/L compared with roots of control wheat seedlings (Figure 3A). FW and DW of roots were also lower under the influence of (Bio)Ag NPs, which was most evident after using a concentration of 40 mg/L (Figure 3B,C). Yanik and Vardar [41,42] studied the response of wheat seedlings to Ag NPs treatment in the concentration range of 0.5–20 mg/L and the size of 10 nm and showed that it was hormetic. This means that Ag NPs at low concentrations (0.5–1 mg/L) stimulated root growth, while at higher concentrations (5–20 mg/L), they had an inhibitory effect. Similar responses to Ag NPs were found in the roots of barley [38,39] and tomato [42,43] seedlings.

#### 2.3.1. The Formation of Reactive Oxygen Species (ROS) in Root Tips

**Figure 4:**
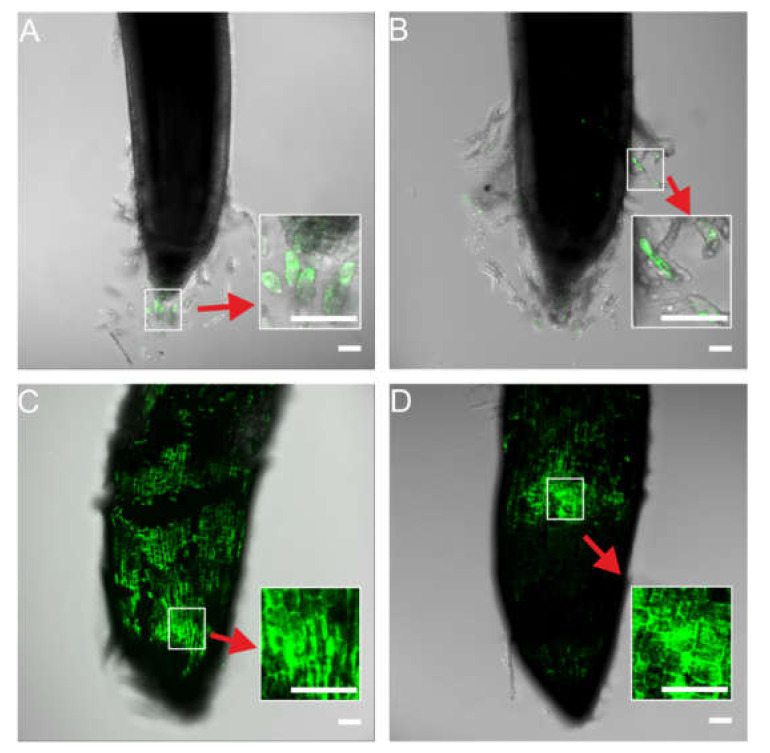
Determination of cellular ROS by H_2_DCF-DA assay (**A**–**D**) in the wheat root tips after 3 days of seedling development in (Bio)Ag NPs at 0 (**A**), 10 (**B**), 20 (**C**), and 40 (**D**) mg/L. Scale: the length of the horizontal white bars equals 100 µm.

2,7-Dichlorodihydrofluorescein diacetate (H_2_DCF-DA) was used as a marker of ROS formation. H_2_DCF-DA is deacetylated by intracellular esterases to H_2_DCFH, which is rapidly oxidized to highly fluorescent DCF by ROS in cells. ROS fluorescence was observed in Ag NPs treated and non-treated control tips (Figure 4). The highest fluorescence intensity was observed in the root tips of seedlings developing in the presence of Ag NPs at concentrations of 20 and 40 mg/L, and the lowest intensity was observed in the control and with (Bio)Ag NPs at a concentration of 10 mg/L (Figure S2, Supplementary Materials). Moreover, fluorescence that indicated ROS formation was observed in root tip cells (Figure 4A,B, red arrows show enlarged images) in wheat seedlings of control and treated with 10 mg/L (Bio)Ag NPs, whereas in the grain and seedling treated with 20 and 40 mg/L (Bio)Ag NPs, it was observed in the apical meristem region (Figure 4C,D, red arrows show enlarged pictures). Increasing ROS formations in epidermal cells cause a peroxidation of membrane lipids [28,34,41,42], membrane damage [41], and cell apoptosis [43,44]. Excess ROS can also stimulate cell wall lignification and callose deposition [44,45]. As a result, increased cell wall rigidity reduces cell elongation and cell wall permeability and inhibits root elongation [46].

#### 2.3.2. Fluorescence Imaging of Cell Death in Root Tips

**Figure 5:**
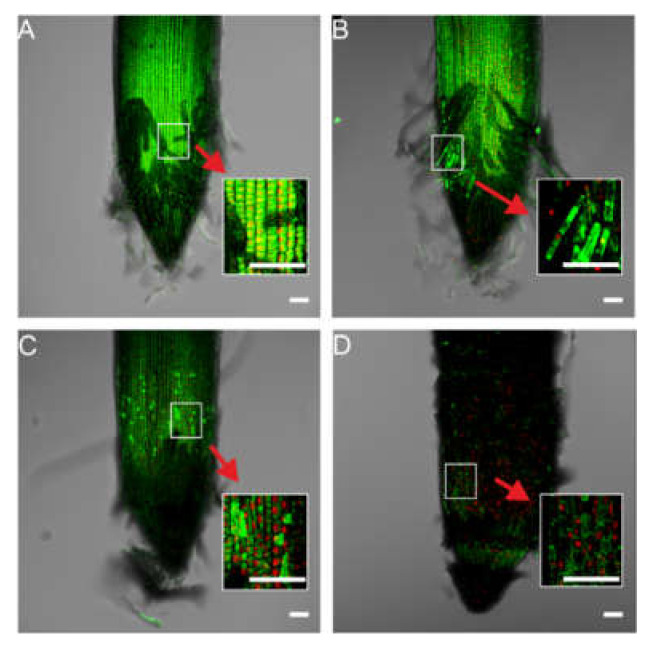
Fluorescence images of a live/dead assay of the wheat root tips after 3 days of seedling development in (Bio)Ag NPs at 0 (**A**), 10 (**B**), 20 (**C**), and 40 (**D**) mg/L. Syto9 green fluorescence is characteristic for viable cells and nonviable cells, and PI red fluorescence is characteristic for nonviable cells only (red arrows show enlarged pictures). Scale: the length of the horizontal white bars equals 100 µm.

The effect of (Bio)Ag NPs on wheat seedling root tips was analyzed using a live/dead fluorescence assay (Figure 5). Root tips showed characteristic green (live cells) and/or red (dead cells) fluorescence. However, when exposed to high concentrations of (Bio)Ag NPs (20 and 40 mg/L), there were fewer live cells in the root tips (Figure 5C,D) compared with the tips of control and low concentration (10 mg/L) (Bio)Ag NPs-treated seedlings (Figure 5A,B).

These results may indicate that as the concentration of biologically synthesized Ag NPs increases, toxic effects are spread from septic cap cells to epidermal and cortical cells of the root apex and elongation zone. The genotoxic effects of Ag NPs, including aberrations in mitosis, nuclear erosion and elongation, and chromosome damage, have been previously documented in wheat [31], faba bean [47], and garden pea [48] seedlings. In our study, increased fluorescence in root tips under (Bio)Ag NPs (Figure 4) indicating ROS generation interacted with increased numbers of dead cells (Figure 5C,D).

The increase in the toxic effects of (Bio)Ag NPs in wheat roots may not only result from the increase in its concentration but also from their relatively small size (5–10 nm) and spherical shape as well as the exposure time. Such relationships have been reported previously in wheat [31]. The small (8 nm) and spherical Ag NPs showed higher antimicrobial activity than larger (45–47 nm) as well as decahedral or triangular Ag NPs [26]. This is related to the fact that small Ag NPs particles can easily pass through pores in the cell walls of wheat root epidermis, enter the plasma membrane, reach the cortex tissues, and be transported by xylem [16,17,26,33,44].

Thus, it might be expected that nanoparticle transport from the roots to the coleoptile should also slow its growth in a concentration-dependent manner, similarly to that in roots. However, our observations did not confirm this, because coleoptile growth was inhibited to the same extent by (Bio)Ag NPs at concentrations of 10 as 40 mg/L (Figure 3). Therefore, other factors such as changes in transpiration/metabolism of phytohormones and increased ROS production [45,46,48,49,50] presumably contribute to this.

### 2.4. Profile and Content of Polar Metabolites in Wheat Seedlings 

The germination process of wheat grains lasted from 24 to 35 h after the imbibition (data not shown). Germination was accompanied by changes in the proteome [50,51,52] and the transcriptome of the embryo and a reactivation of cellular metabolism [52,53,54]. The initiation of degradation of the main energy reserves includes the following: starch and proteins in the scutellum, and the endosperm and aleurone layer precedes the start of respiration. As a result of metabolic changes, soluble carbohydrates and amino acids are released, providing energy, carbon skeletons, and nitrogen to developing seedlings required for their transition from heterotrophic to autotrophic plant growth [52,53,54,55]. In our study, the qualitative and quantitative composition of polar metabolites identified in wheat seedlings illustrates the metabolic status on the second day after the activation of cellular metabolism.

**Figure 6:**
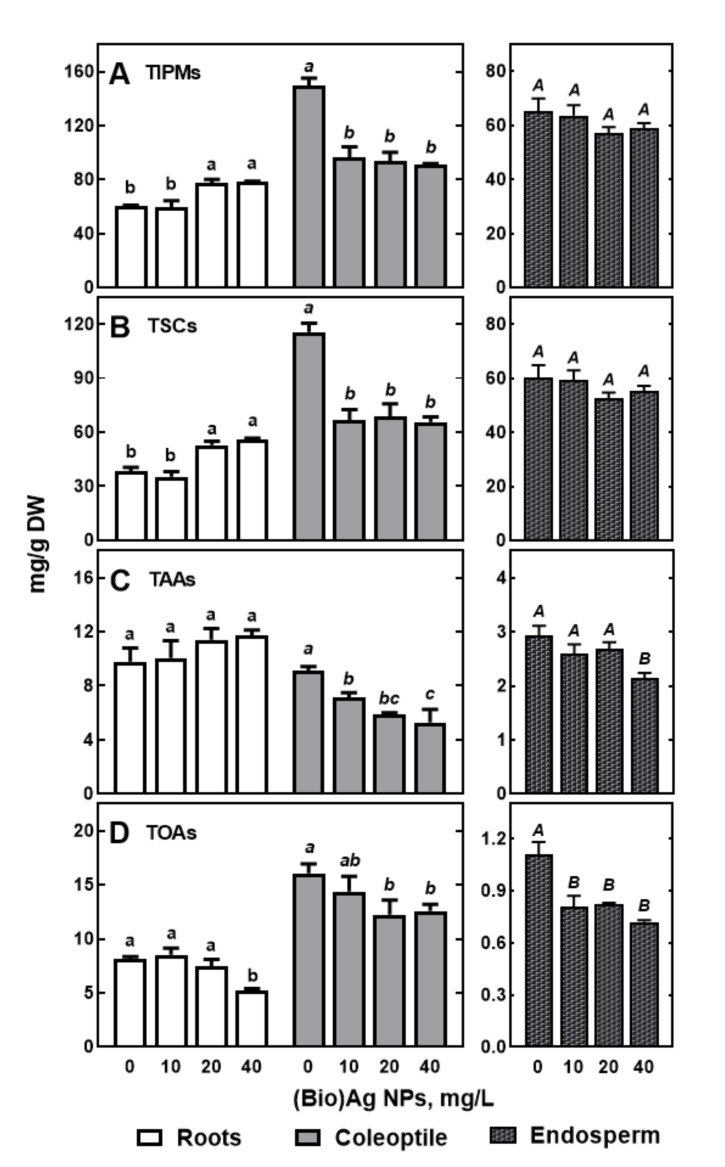
The effect of (Bio)Ag NPs on the concentration of total identified polar metabolites ((**A**), TIPMs), including total soluble carbohydrates ((**B**), TSCs), total amino acids ((**C**), TAAs), and total organic acids ((**D**), TOAs) in roots, coleoptile, and endosperm of 3-days-old seedlings of wheat (*Triticum aestivum* L. cv. ‘Ostka Strzelecka’). Control grains germinated in dd. water. Values are means (*n* = 3) ± SD. Bars with the same letters (a–b, *a–c*, *A–B*) are not significantly (*p* < 0.05) different after the ANOVA test and Tukey’s post hoc corrections.

In wheat seedlings, 32 polar metabolites were identified. These include soluble carbohydrates (8), amino acids (17), organic acids (6), and others (urea and phosphoric acid, Table S1, Supplementary Materials). Most of them were present in all seedling tissues except urea and maltotriose, which were detected only in the endosperm (Tables S2–S4, Supplementary Materials). In the coleoptile of control seedlings, the concentration of total identified polar metabolites (TIPMs) was more than 2-fold higher (149.63 mg/g DW) than in roots and the endosperm (60.41 and 65.30 mg/g DW, respectively). Similarly, the total soluble carbohydrate (TSC) concentration was the highest in coleoptile, lower in the endosperm, and the lowest in seedlings roots (115.58, 60.24, and 38.92 mg/g DW, respectively). Furthermore, total organic acid (TOA) levels were 2-folds higher in coleoptile than in roots (16.06 and 8.35 mg/g DW, respectively) and much higher than in the endosperm (Figure 6).

The substantially higher content of osmolytes (sugars and organic acids) in the coleoptile than in the roots may have contributed to the maintenance of the water potential gradient between these organs, which favored the supply of water to the shoot. Moreover, the coleoptile contained significantly more phosphoric acid than the roots and endosperm (8.85, 4.02, and 1.01 mg/g DW, respectively). This was presumably a result of differences in phosphate translocation into roots/shoot plants from the endosperm and scutellum, where they are synthesized by phytate hydrolysis [55,56] and/or increased amounts of phosphorylated metabolites, phospholipids, proteins, and nucleotides in faster-growing roots. It is interesting that the concentration of total amino acids (TAAs) in roots and coleoptile was almost equal (9.79 and 9.12 mg/g DW) and 3-fold higher than that in the endosperm (2.94 mg/g DW). This indicates that mobilization of storage proteins, beginning during grain germination [51,52], continued to support the nitrogen requirements of growing seedlings.

(Bio)Ag NPs at concentrations of 20 and 40 mg/L markedly increased the content of TIPMs in roots while being decreased in coleoptile and endosperm (Figure 6A). The content of TIPMs in the coleoptile decreased, regardless of (Bio)Ag NPs concentration. However, soluble carbohydrates were accumulated in the roots (Figure 6B). In endosperm, the levels of total amino acids (TAAs) and TOAs declined (Figure 6C,D). Presumably, drastic changes in tissues metabolism by using Ag NPs are closely related to seedling growth and development, as it was found earlier after the foliar application of copper and manganese NPs on 3-week-old wheat plants [56,57], as well as in Ag NPs treated Arabidopsis thaliana [57,58]. The results of PCA analysis of our results, confirm that (Bio)Ag NPs considerably affected profiles and metabolite contents in tissues of roots and coleoptile.

### 2.5. Changes in Metabolic Profiles of Polar Compounds under the Influence of Ag NPs

**Figure 7:**
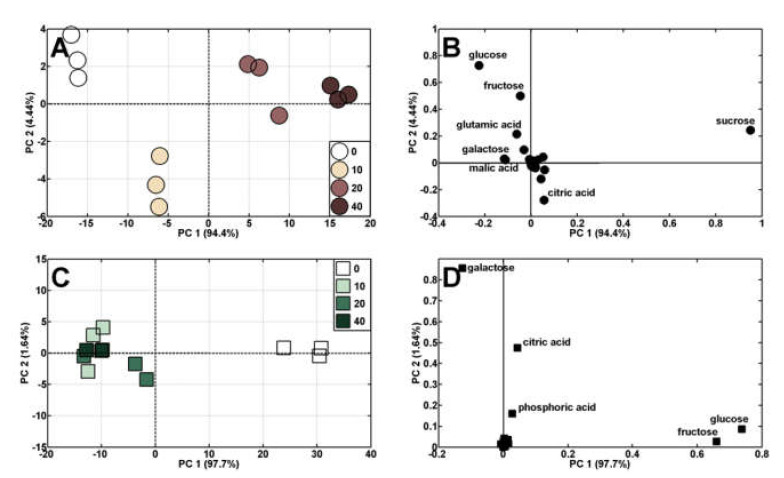
PCA profiles of roots (**A**) and coleoptile (**C**) metabolites of wheat seedlings developing in the presence of (Bio)Ag NPs at concentrations 0 (control), 10, 20, and 40 mg/L and PCA loading plots of the polar metabolites (**B**,**D**).

Principal components analysis (PCA) of polar metabolites in wheat seedling tissues showed a shift in the distribution of results obtained for roots and coleoptiles (Figure 7) and, to a lesser extent, the endosperm (Figure S3, Supplementary Materials).

The analytical results of control roots and those treated with (Bio)Ag NPs at 10 mg/L were to the left of PC1, sharing 94.4% of the variability, whereas root samples developing at higher (Bio)Ag NPs concentrations (20 and 40 mg/L) were to the right (Figure 7A). This distribution of results resulted from changes in sucrose and monosaccharide concentrations (glucose, fructose, and galactose), as well as malic and citric acid and glutamate (Figure 7B).

All results analysis of coleoptile samples from seedlings treated with (Bio)Ag NPs were to the left of PC1, sharing 97.7% of the variability, while the control was to the right (Figure 7C). This was determined by changes in glucose, fructose, galactose, citrate, and inorganic phosphate concentration (Figure 7D). The PCA of metabolites in the endosperm showed that the distribution of samples was influenced by changes in concentrations of glucose, maltose, and maltotriose, which are products of starch hydrolysis and were located to the right of PC1 (sharing 92.9% of the variation) and sucrose (Figure S3, Supplementary Materials).

#### 2.5.1. The Soluble Carbohydrate Contents in Wheat Seedlings and Their Changes upon Exposure to Ag NPs

**Figure 8:**
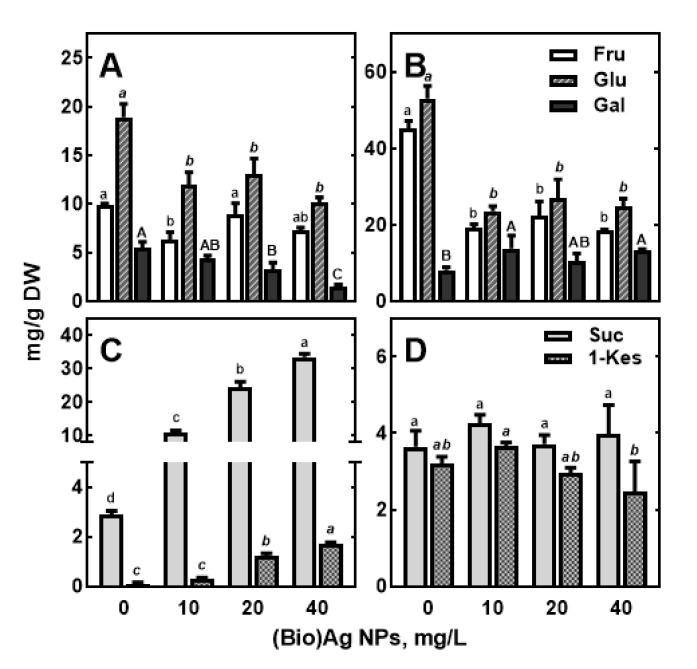
The effect of (Bio)Ag NPs on fructose (Fru), glucose (Glu), galactose (Gal), sucrose (Suc), and 1-kestose (1-Kes) contents in roots (**A**,**C**) and coleoptile (**B**,**D**) of 3-days-old seedling of wheat (*Triticum aestivum* L. cv. ‘Ostka Strzelecka’). Values are means (*n* = 3) ± SD. Bars marked with the same letters are not significantly (*p* < 0.05) different when calculated with ANOVA and Tukey’s post hoc test.

In the organs of wheat seedlings, soluble carbohydrates were the major quantitative compounds among polar metabolites (Figure 6). Their contribution ranged from 63.71 and 77.24% TIPM in roots and coleoptile, respectively, to 92.25% in the endosperm. These results confirm the crucial role of soluble sugars as a source of carbon skeletons and energy during early seedling development [58,59]. The major carbohydrates were glucose, fructose and galactose in both roots (18.90, 9.89, and 5.49 mg/g DW) and coleoptile (53.01, 45.04, and 8.17 mg/g DW), while maltose, sucrose, and glucose (34.22, 10.80 and 9.94 mg/g DW) were the major carbohydrates in the endosperm (Tables S2–S4, Supplementary Materials). The high levels of glucose, fructose, galactose, and sucrose in the growing tissues (Figure 8) indicate both the mobilization of the starchy endosperm [59,60,61] and the transport of the released sugars to growing seedlings [61,62].

The higher concentrations of sucrose and monosaccharides in control seedlings than in endosperm (Tables S2–S4, Supplementary Materials) confirmed the activation of enzymes responsible for sugar metabolism, such as sucrose-phosphate synthase and sucrose phosphatase [62,63], which synthesize sucrose in the scutellum by degrading starch [61,62]. Invertase and sucrose synthases that hydrolyze sucrose in roots and coleoptile are also involved in this process [63,64,65]. In addition, glucose and maltose, which are the end products of starch degradation in the endosperm, are converted in the scutellum to sucrose, which is transported to the growing roots and coleoptiles [61,62]. Thus, the 10-fold higher concentration of monosaccharides than sucrose in roots and coleoptile (Figure 8), as the only sink tissues, may be due to high invertase and/or sucrose synthase activity [62,63,64,65]. Sucrose synthase activity has been shown to correlate with cellulose deposition in wheat roots [64,65]. High glucose levels appear to be important for the development of the entire root system as well as lateral root primordia [63,64].

In roots of wheat seedlings developing in Ag NPs suspensions, glucose and galactose concentrations decreased compared with the control (Figure 8A, Table S2, Supplementary Materials), whereas sucrose and 1-kestose contents increased 10-fold (Figure 8C). In the coleoptile, glucose, as well as fructose levels, decreased approximately 2-fold, independent of (Bio)Ag NPs concentration (Figure 8B), whereas galactose content increased (Table S3, Supplementary Materials). Moreover, coleoptile seedlings developing in the presence of concentrated biologically synthesized Ag NPs suspension (40 mg/L) showed a significant decrease in 1-kestose but not sucrose (Figure 8D). In contrast, there was a slight downward trend in the levels of most carbohydrates (maltotriose, maltose, glucose, fructose, galactose, and *myo*-inositol) in the endosperm as (Bio)Ag NPs increased, except for sucrose and 1-kestose, which increased slightly (Table S3, Supplementary Materials). The results suggest that in seedlings developing in the presence of (Bio)Ag NPs, there is both a reduction in starch mobilization in the endosperm and a reduction in the utilization of released sugars. The inhibitory effect of Ag NPs on starch hydrolyzing α-amylase in cereal grains was documented by Johnson et al. [65,66], while a stimulatory effect was found in germinating aged rice seeds [66,67]. Other nanoparticles, such as ZnO NPs [67,68], and heavy metals, such as lead [68,69], can also inhibit the activity of this enzyme. Currently, it remains to be resolved which forms of α-amylase, among the five present in the endosperm of germinating wheat grains [60,61,69,70], are susceptible to Ag NPs or silver ions released from Ag NPs after its uptake by roots [34,39,40]. Furthermore, the effects of Ag NPs and Ag^+^ on the activity of enzymes catalyzing sucrose hydrolysis are also unknown.

The accumulation of sucrose in roots under (Bio)Ag NPs found in this study was presumably the cause of root tip thickening. Indeed, increasing sucrose content may have increased the osmotic potential of the tissues and consequently increased water uptake without causing root elongation. On the other hand, the accumulation of sucrose and the decrease in glucose content could also be the result of either an impairment of invertase and/or sucrose synthase activity [63,64,65] or the use of glucose as a substrate for respiration, accelerated under stress conditions [70,71].

The inhibition of coleoptile growth seems to be associated with an increased concentration of galactose (from 8.17 to ca 13 mg/g DW), a known inhibitor of coleoptile growth, affecting the synthesis of UDP-glucose, a sole substrate for cellulose synthesis [72]. In roots opposite to coleoptile, galactose contents decreased with increasing (Bio)Ag NPs concentrations (Figure 8A).

#### 2.5.2. The Amino Acids Contents in Wheat Seedlings and Their Changes upon Exposure to (Bio)Ag NPs

In germinating cereal grains, the mobilization of storage proteins begins in the embryo and scutellum. It then occurs in the aleurone cell layer, where de novo synthesized hydrolases are secreted into the starchy endosperm to mobilize storage proteins [72,73]. Amino acids released from the proteins of the starchy endosperm and aleurone layer [50,51,52] are taken up by scutellum through various systems. In the scutellum of 4-day-old wheat seedlings, glutamine is taken up by two nonspecific amino acid uptake systems in which one is inhibited and the other is not inhibited by asparagine [73,74].

The major amino acid in the roots of control wheat seedlings was glutamic acid while it was asparagine in the coleoptile (2.43 and 1.95 mg/g DW, respectively), while in the endosperm was proline (0.59 mg/g DW). The high contents of glutamate/glutamine and aspartate/aspartate compared to other amino acids (Tables S2–S4, Supplementary Materials) confirm the important role of both glutamate and aspartate pathways [74,75,76] in wheat seedling metabolism. Thus, their role as major amino acids translocated through the phloem from source tissues to wheat sink tissues is also confirmed [76,77]. In the phloem sap of mature wheat plants, the major amino acids are glutamate and aspartate [76,77]. The catabolism of these amino acids and others released from storage proteins is important for the synthesis of new proteins and other nitrogenous compounds during early seedling development [77,78]. The contents of aspartate and glutamate shown in wheat seedling roots exceeded those of the amide forms, while the opposite pattern was found in coleoptile (Tables S2 and S3, Supplementary Materials).

**Figure 9:**
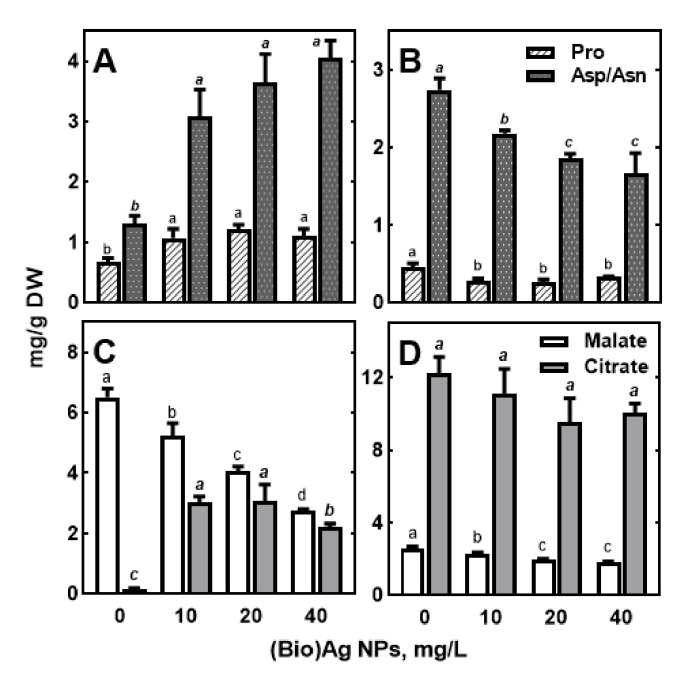
The effect of (Bio)Ag NPs on the content of proline (Pro), aspartate/asparagine (Asp/Asn), malate, and citrate in roots (**A**,**C**) and coleoptile (**B**,**D**) of 3-days-old seedling of wheat (*Triticum aestivum* L. cv ‘Ostka Strzelecka’). Control grains germinated in dd. water. Values are means (*n* = 3) ± SD. Bars with the same letters (a–c, *a–c*) are not significantly (*p* < 0.05) different after the ANOVA test and Tukey’s post hoc corrections.

The effect of (Bio)Ag NPs on the content of total amino acids (TAAs) in roots was insignificant (Figure 2C), but in some of them, such as proline, aspartate/asparagine were accumulated under the resulting stress (Figure 9A). Similar changes occurred for hydroxyproline, valine, and leucine, but these were present at much lower concentrations (Table S2, Supplementary Materials).

The opposite trend of proline and aspartate/asparagine accumulation was found in the coleoptile compared to that in the roots (Figure 9B). Proline accumulation in roots of Ag NPs/Ag^+^-treated plants has been shown previously both in wheat [79] and in other species [14]. Moreover, oxidative stress in wheat induced by metal cations such as copper [79,80] and nickel [80,81] also causes proline accumulation. Thus, the suggested protective roles of this amino acid against abiotic stresses by scavenging ROS and as a factor maintaining redox balance under stress conditions, as well as a stabilizer of cell structures and enzymes, seem to be important also in the response of wheat seedlings to Ag NPs [81,82,83]. In response to oxidative stress, proline can be converted non-enzymatically to gamma-amino butyric acid (GABA) [83,84]. In our study, it was observed that the level of GABA under (Bio)Ag NPs did not change in roots but decreased in coleoptile (Tables S2 and S3, Supplementary Materials).

It is important to note that in the roots of wheat seedlings, an increase in aspartate/asparagine (Figure 9A) was accompanied by a decrease in glutamate/glutamine concentration (Table S2, Supplementary Materials). In endosperm, the contents of all amino acids decreased slightly with increasing concentrations of (Bio)Ag NPs (Table S4, Supplementary Materials). However, a common response of roots and coleoptiles to Ag NPs appeared to be a decrease in the contents of aromatic amino acids, phenylalanine, and tryptophan (Tables S2 and S3, Supplementary Materials). Some data indicated that stimulation of secondary metabolism by (Bio)Ag NPs results in the accumulation of phenolic compounds [85]

#### 2.5.3. The Effect Ag NPs on Contents of Organic Acids and Other Compounds in Wheat Seedlings

The activation of metabolism in germinating seeds and subsequent seedling growth and development depends on the efficiency of respiration providing energy and organic acids [60,61,62]. Among tricarboxylic acids (TCAs), only citrate, malate, and fumarate were present at detectable levels in wheat seedlings (Tables S2–S4, Supplementary Materials). In (Bio)Ag NPs-treated roots, the concentration of malate, which was the major TCA, decreased approximately 2-fold (from 6.51 to 2.74 mg/g DW, Figure 9C). In contrast, the contents of citrate and fumarate enhanced from 0.16 to 2.22–3.06 and from 0.03 to 0.05 mg/g DW, respectively, as the concentration of (Bio)Ag NPs increased (Table S2). In coleoptile containing 2-fold more TCAs than roots (16.06 and 8.01 mg/g DW, respectively, Figure 2D), all occuring organic acids decreased (Figure 9D). In the endosperm, TCAs were present at several-fold lower levels compared with seedling tissues, and their changes were small, and only citrate and lactate levels were significantly reduced (Table S4, Supplementary Materials).

The reason for the ca. 40-fold lower level of citrate than malate in roots, in contrast to the ca. 6-fold higher level of citrate than malate in coleoptile and endosperm, is not known. Citrate and oxalate are known to be secreted from roots into the soil solution in the rhizosphere, thus allowing efficient phosphorus mobilization [85,86,87]. However, such a role during early seedling development seems unlikely due to the endogenous mobilization of phytate [55,56]. On the other hand, the content of phosphoric acid in roots was found to be 2-times lower than in coleoptile. In the seedlings developing in the presence of Ag NPs, the concentration of phosphoric acid increased in roots from 4.01 to 5.78 mg/g DW, while it decreased in coleoptile and endosperm from 8.85 to 7.68 and from 1.01 to 0.72 mg/g DW, respectively. Interactions between phosphates and Ag NPs are possible [87,88], but the presence of interactions in tissues or on the cell surface is unknown.

## 3. Materials and Methods

### 3.1. Bio-Synthesis and Characteristics of Ag NPs 

In the present study, silver nanoparticles have been synthesized according to the method described in the previous study [37,88] using a *Lactobacillus paracasei ssp. paracasei DSM 2649 DSM* isolate as a source. The size and shape of the Ag NPs were analyzed with transmission electron microscopy (TEM) at 80 kV (JEM-1400, JEOL, Tokyo, Japan), while elemental composition was carried out by an Energy Dispersive X-Ray spectrometer operated with a transmission electron microscopy (FEI Tecnai F20 X-Twintool, FEI Europe, Frankfurt/Main, Germany). The size distribution was calculated with the ImageJ program (Version 1.53k) [89].

#### Preparation of Ag NPs Suspensions

The Ag NPs stock suspension (200 mg/L) was prepared (at the day of phytotoxicity test) by the addition of 23.6 mg of dry Ag NPs to 118 mL of double-distilled water (DDW) and sonication (Sonic-3, 310 W, 40 KHz, POLSONIC Pałczyński, Warsaw, Poland) of suspension for 30 min. Then, the stock suspension was diluted with DDW to obtain Ag NPs at concentrations 10, 20, and 40 mg/L.

### 3.2. Preliminary Experiment on Seed Germination

For the determination of phytotoxicity of (Bio)Ag NPs to wheat (*Triticum aestivum* L.) grains and seedlings, two winter cultivars ‘Jantarka’ and ‘Ostka Strzelecka’ (purchased from DANKO and Hodowla Roślin Strzelce, Poland) and one spring cultivar ‘Collada’ (purchased from KWS Lochow Polska sp. z o.o, Prusy, Poland) were examined. The grains were germinated in Petri dishes (120 × 20 mm) containing 15 mL of DDW (control) or Ag NPs suspensions (at the concentrations of 20 and 40 mg/L) at 20 °C in the dark. Each treatment was made in four replicates (each replicate is one Petri dish containing 40 grains). After 3 days of incubation, germinability (G, %) was calculated as follows in Equation (1):

(1) $$G=\frac{\mathrm{number}\;\mathrm{of}\;\mathrm{germinated}\;\mathrm{grains}}{\mathrm{total}\;\mathrm{number}\;\mathrm{of}\;\mathrm{incubated}\;\mathrm{grains}}\;x\;100\%$$

where the germinated grains where those in which growing embryo ruptured testa. Moreover, the percentage of developing seedlings (DS, %) was calculated as follows in Equation (2):

(2) $$\mathrm{DS}=\frac{\mathrm{number}\;\mathrm{of}\;\mathrm{seedlings}\;\mathrm{with}\;\mathrm{coleoptile}\;\mathrm{and}\;3\;\mathrm{roots}\;\left(\mathrm{length}\;\geq 5\mathrm{mm}\right)}{\mathrm{number}\;\mathrm{of}\;\mathrm{germinated}\;\mathrm{grains}}\;\times \;100\%$$

The longest radicle length and coleoptile length of developing seedlings (from three replicates) were measured, and the seedlings then were separated into coleoptile (including plumule), roots (including scutellum and mesocotyl), and endosperm (with seed coat). The tissues were weighed and dried at 80 °C for 20 h. The fresh weight (FW) and dry weight (DW) were expressed in mg per roots, coleoptile, and endosperm.

### 3.3. Phytotoxicity of (Bio)Ag NPs to Wheat Seedlings

Based on the results of the above experiment, focusing on the comparison of biologically synthesized Ag NPs influence on germination and development of seedlings of three wheat cultivars, the cultivar ‘Ostka Strzelecka’ was selected for physiological and metabolomic analyses of (Bio)Ag NPs phytotoxicity. The grains of this wheat variety were germinated in DDW (control) and 15 mL of (Bio)Ag NPs suspension at concentrations 10, 20, and 40 mg/L (40 grains per each of 6 replicates). After the measurement of root and coleoptile length and FW of roots, coleoptile, and endosperm (separately, from three replicates), tissues were frozen in liquid nitrogen, stored in an ultra-freezer (at −76 °C) for 2 days, and freeze-dried for 48 h (shelf freeze-dryer, Alpha 1–2 LD, Martin Christ, Osterode am Harz, Germany). The water concentration (WC) was calculated as the difference between the fresh weight (FW) and dry weight (DW) and expressed as a percentage of FW. The freeze-dried and pulverized tissues were taken for metabolite profiling.

#### 3.3.1. Microscopic Analyses

The morphology of 3-days-old seedlings (from 3 remaining replicates) was documented by using 3D VHX-7000 Digital Microscope (KEYENCE, Mechelen, Belgium).

#### 3.3.2. Generation of Reactive Oxygen Species and Cytotoxicity

The 0, 10, 20, and 40 mg/L (Bio)Ag NPs treated root tips of wheat were placed in 2,7-dichlorodihydrofluorescein diacetate (H_2_DCF-DA; Sigma-Aldrich, Poznan, Poland) to detect reactive oxygen species (ROS). The samples were placed in a 10 μM solution of H_2_DCF-DA in 0.1 M phosphate-buffered saline (PBS, pH = 7.4) in the dark. After 30 min, H_2_DCF-DA was replaced with fresh PBS for another 30 min [90] and imaged by confocal laser scanning microscopy (CLSM).

The new fresh-cut wheat root tips were also placed on microscope slides and stained with the mix of SYTO^®^ 9 and propidium iodide (PI) stain (LIVE/DEAD, L7007, Life Technologies, Carlsbad, CA, USA). The PI solution was used to identify nonviable cells within the root tip [91], and SYTO^®^ 9 was used to identify nonviable and viable cells [92]. The samples were stained for 15 min in the dark, rinsed in water, and imaged by CLSM. The presented images were z-stacked, and quantitative analyses of fluorescence intensity were performed by CLSM (Leica TCS SP5, Leica Microsystems, Wetzlar, Germany) and Leica Application Suite 2.0.2 build 2038. The following excitation and emission wavelengths were used in the experiment:

- For ROS analysis—λex 488 nm and λem 515–565 nm;
- For cytotoxicity analysis—for SYTO^®^ 9 staining λex = 488 nm and λem = 500–520 nm; for propidium iodide (PI), λex 561 nm and λex 610–650 nm.

### 3.4. Metabolite Profiling

Dry tissues were pulverized in a mixed mill, and polar metabolites were extracted using the method described earlier [93]. Briefly, polar metabolites were extracted from 30 to 40 mg of dry tissues with 50% methanol at 70 °C for 30 min (with continuous shaking, 500 rpm). The non-polar compounds were removed by cold chloroform, and the polar fraction was evaporated to dryness in a speed vacuum rotary evaporator (JW Electronic, Warsaw, Poland). The derivatization of the metabolites involved the use of O-methoxamine hydrochloride (at 37 °C for 75 min, with continuous shaking, 500 rpm) and then a mixture of MSTFA (*N*-methyl-*N*-trimethylsilyl-trifluoroacetamide) with pyridine (1:1, *v*/*v* ratio, at 70 °C for 30 min), according to Lisec et al. [94]. The mixtures of TMS-derivatives were separated on a ZEBRON ZB-5MSi Guardian capillary column (Phenomenex, Torrance, CA, USA) in a gas-chromatograph coupled with a quadrupole mass spectrometer (QP-GC-2010, Shimadzu, Kyoto, Japan). Metabolites were identified by a comparison of retention time (RT), relative retention time (RRT), retention indices (RI), and mass spectra obtained for original standards (Sigma-Aldrich, Saint Louis, MO, USA) and from the NIST 05 library (National Institute of Standards and Technology, Gaithersburg, MD, USA). The concentration of identified polar metabolites was calculated according to the method described previously [93].

### 3.5. Statistics

The results were subjected to one-way ANOVA with a post hoc Tuckey’s test (at the probability level *p* < 0.05) using Statistica software (version 12.0; StatSoft, Tulsa, OK, USA). Graphs were prepared using GraphPad Prism (version 8.0; GraphPad Software, San Diego, CA, USA). Principal Component Analysis (PCA) was performed in the COVAIN program [95], using MATLAB software (version 2013a, Math Works, Natick, MA, USA), in order to compare the effect of Ag NPs on metabolic profiles of roots, coleoptile, and endosperm of 3-days-old wheat seedlings.

## 4. Conclusions

The presence of low molecular weight polar metabolites at considerable levels (Figure 6) in the roots, coleoptile, and endosperm of 3-days-old wheat seedlings (Tables S3–S5, Supplementary Materials) confirms early storage mobilization in the endosperm/aleurone layer and the transport of released sugars/amino acids to growing seedlings. Moreover, the metabolic profiles of control wheat seedlings confirm earlier reports on changes in sugars and amino acids in germinating wheat. The typical features of this profile seem to be the following: (i) the high concentration of monosaccharides (glucose and fructose) in root and coleoptile, while maltose and sucrose are observed in the endosperm; (ii) quantitative domination of asparagine, glutamic acid, and proline among protein amino acids; and (iii) the emergence and accumulation of GABA. The obtained results confirm that during the early development of wheat seedlings, the activity of various hydrolytic enzymes (amylases, proteases, glucanases, and others) increases. The products of the degradation of starch in the endosperm, proteins, and phytate in the aleurone layer sugars, amino acids, *myo*-inositol, phosphate, and minerals are translocated through the scutellum into growing roots and coleoptile.

Biologically synthesized Ag NPs applied at a concentration of 20 or 40 mg/L caused a growth inhibition of wheat seedlings. This was accompanied by the formation of a brown coloration of root caps and fluorescence indicating the production of reactive oxygen species. When the concentration of Ag NPs was 40 mg/L, browning spread from the meristematic zone to the elongation zone. In addition, (Bio)Ag NPs caused drastic metabolic changes in wheat tissues. The inhibition of coleoptile growth appears to be related to elevated galactose concentrations. The content of most metabolites decreased under the influence of (Bio)Ag NPs in the coleoptile and endosperm, in contrast to the roots of wheat seedlings. Changes in the content of polar metabolites in seedlings under the influence of (Bio)Ag NPs indicate a disruption of primary metabolism, the mobilization of starch and reserve proteins, and the translocation of sugars and amino acids from the endosperm to the tissues of developing seedlings.

## Figures and Tables

**Table 1:** The effect of (Bio)Ag NPs on germinability, percentage of developing seedlings, length of primary seminal root and coleoptile, and the fresh and dry weights of roots, coleoptile (including covered shoot), and endosperm (including pericarp) in 3-day-old seedlings of wheat cultivars. Values are means (*n* = 4). The same superscript letters by the values indicate no significant (*p* < 0.05) differences according to the ANOVA test and post hoc Tukey’s corrections (calculated for each column and cultivar separately). Developing seedlings means seedlings with normal coleoptile and one-two pair of seminal roots each at least 3–4 mm long.

	(Bio)Ag NPs Concentration (mg/L)		Cultivar	
		Ostka Strzelecka	Jantarka	Collada
Germinability (%)	0	93.33 ^a^	86.67 ^a^	92.50 ^a^
	20	95.83 ^a^	90.83 ^a^	95.83 ^a^
	40	95.00 ^a^	85.00 ^a^	97.50 ^a^
Developing seedlings (%)	0	63.33 ^a^	43.33 ^a^	74.17 ^a^
	20	57.50 ^a^	40.83 ^a^	55.83 ^a^
	40	49.17 ^a^	67.50 ^a^	64.17 ^a^
Primary seminal root length (mm)	0	29 ^a^	31 ^a^	48 ^a^
	20	27 ^b^	20 ^b^	34 ^b^
	40	15 ^c^	11 ^c^	23 ^c^
Coleoptile length (mm)	0	17 ^a^	19 ^a^	30 ^a^
	20	13 ^b^	17 ^ab^	28 ^a^
	40	13 ^b^	14 ^b^	23 ^b^
Roots fresh weight (mg)	0	24.8 ^a^	24.8 ^a^	46.6 ^a^
	20	20.9 ^b^	20.2 ^b^	37.6 ^ab^
	40	19.9 ^c^	13.5 ^c^	28.4 ^b^
Coleoptile fresh weight (mg)	0	46.6	46.6	38.4 ^a^
	20	37.6 ^ab^	37.6 ^ab^	36.2 ^a^
	40	28.4 ^b^	28.4 ^b^	30.5 ^a^
Endosperm fresh weight (mg)	0	69.4 ^ab^	74.4 ^a^	64.0 ^a^
	20	74.7 ^a^	73.6 ^a^	59.8 ^a^
	40	73.7 ^b^	75.6 ^a^	64.5 ^a^
Roots dry weight (mg)	0	2.3 ^a^	2.1 ^ab^	4.3 ^a^
	20	2.2 ^b^	2.4 ^a^	4.3 ^a^
	40	1.8 ^b^	1.7^b^	4.0 ^a^
Coleoptile dry weight (mg)	0	2.5 ^a^	2.9 ^a^	3.6 ^a^
	20	2.0 ^b^	2.6 ^a^	3.4 ^a^
	40	2.1 ^b^	2.3 ^a^	3.3 ^a^
Endosperm dry weight (mg)	0	37.8 ^a^	40.0 ^a^	30.9 ^a^
	20	37.4 ^a^	39.1 ^a^	29.2 ^a^
	40	37.0 ^a^	40.1 ^a^	32.4 ^a^

## Data Availability

Not applicable.

## Supplementary Materials

The following are available online at https://www.mdpi.com/article/10.3390/molecules27072303/s1, Figure S1. The effect of Ag NPs at 0, 20 and 40 mg/L on the morphology of 3-days-old seedlings of wheat cultivars ‘Ostka Strzelecka’ (A), ‘Jantarka’ (B) and ‘Collada’ (C), Figure S2. The fluorescence intensity of DCF in root tips of 3-days-old seedlings of wheat cv. ‘Ostka Strzelecka’ developing in Ag NPs suspension at 0, 10, 20 and 40 mg/L, Figure S3. PCA of metabolic profiles of the endosperm of 3-days-old seedlings of wheat cv. ‘Ostka Strzelecka’ developing in Ag NPs suspension at 0, 10, 20 and 40 mg/L, Table S1. The parameters of polar metabolites identification in GC-MS analyses. RT—retention time, RRT—relative retention time, RI—retention index, ID—type of identification of each metabolite—using mass spectra of original standards (S) or from NIST 05 library only (L), P—the percentage of similarity of identified metabolite to the standard or metabolite from the library, Table S2. The concentration of total identified polar metabolites (TIPMs), including total soluble carbohydrates (TSCs), total amino acids (TPAAs), total organic acids (TOAs), and total remaining compounds (TRCs) in roots of 3-day-old seedlings of wheat (*Triticum aestivum* L., cv. ‘Ostka Strzelecka’) developing in presence of (Bio)Ag NPs at different concentrations. Means of 3 replicates. The same letters by the values indicate no statistically significant differences (*p* < 0.05) based on ANOVA analysis and Tukey’s post- hoc corrections, Table S3. The concentration of total identified polar metabolites (TIPMs), including total soluble carbohydrates (TSCs), total amino acids (TPAAs), total organic acids (TOAs), and total remaining compounds (TRCs) in coleoptile of 3-day-old seedlings of wheat (*Triticum aestivum* L., cv. ‘Ostka Strzelecka’) developing in presence of (Bio)Ag NPs at different concentrations. Means of 3 replicates. The same letters by the values indicate no statistically significant differences (*p* < 0.05) based on ANOVA analysis and Tukey’s post- hoc corrections, Table S4. The concentration of total identified polar metabolites (TIPMs), including total soluble carbohydrates (TSCs), total amino acids (TPAAs), total organic acids (TOAs), and total remaining compounds (TRCs) in endosperm of 3-day-old seedlings of wheat (*Triticum aestivum* L., cv. ‘Ostka Strzelecka’) developing in presence of (Bio)Ag NPs at different concentrations. Means of 3 replicates. The same letters by the values indicate no statistically significant differences (*p* < 0.05) based on ANOVA analysis and Tukey’s post-hoc corrections.

## Sample Availability

Samples of the compounds are available from the authors.
