# Supplementary material for: The Effect of Bio-Synthesized Silver Nanoparticles on Germination, Early Seedling Development, and Metabolome of Wheat (Triticum aestivum L.)
Source: Molecules. 2022 Apr 1;27(7):2303. doi: 10.3390/molecules27072303 (PMC9000288; doi:10.3390/molecules27072303)
Supplement: Supplementary file 1 [file molecules-27-02303-s001.zip › Supplementary_Figures_S1-S3.pdf]

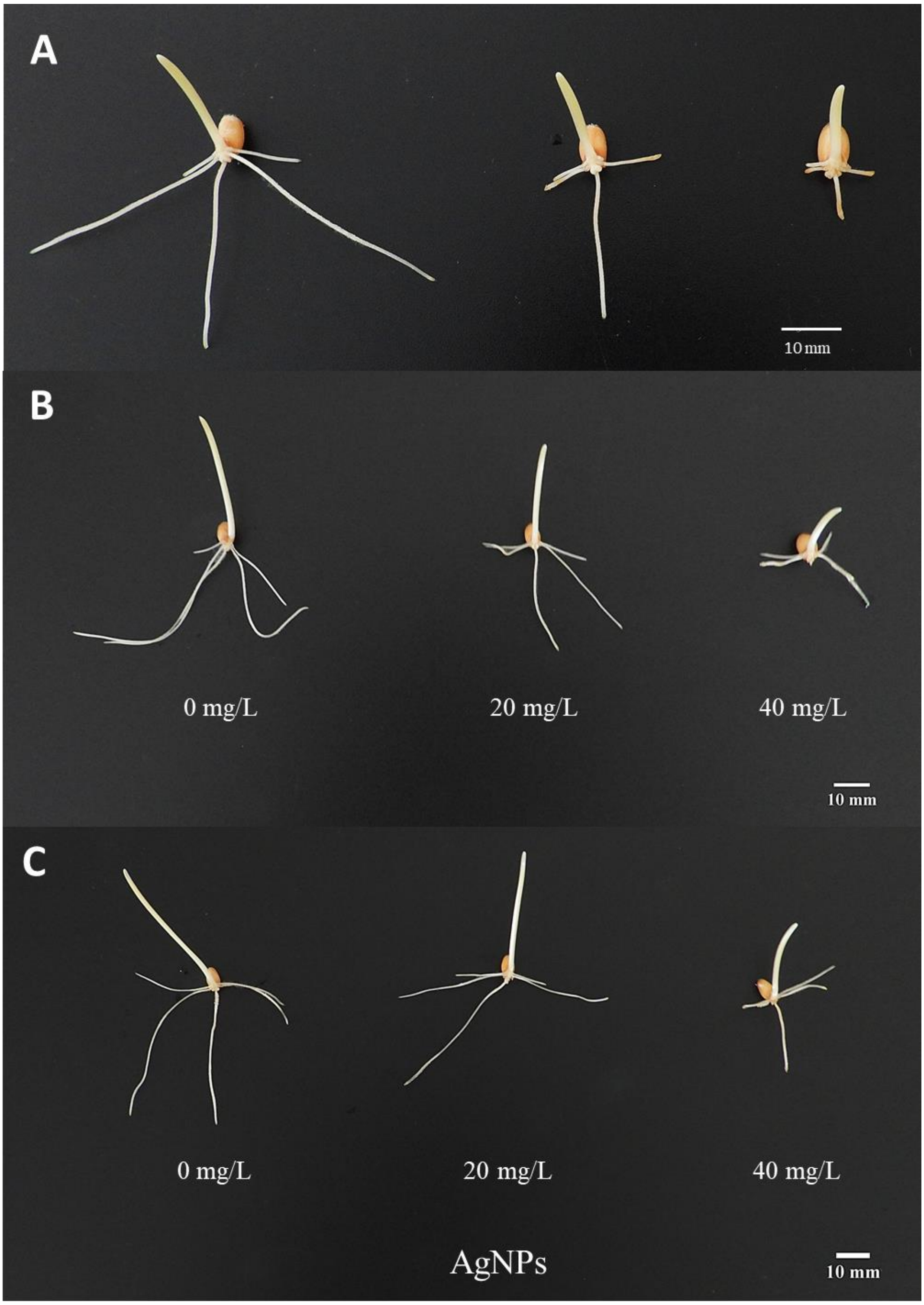

Figure S1. The effect of Ag NPs at 0, 20 and 40 mg/L on the morphology of 3-days-old seedlings of wheat cultivars ‘Ostka Strzelecka’ (A), ‘Jantarka’ (B) and ‘Collada’ (C).

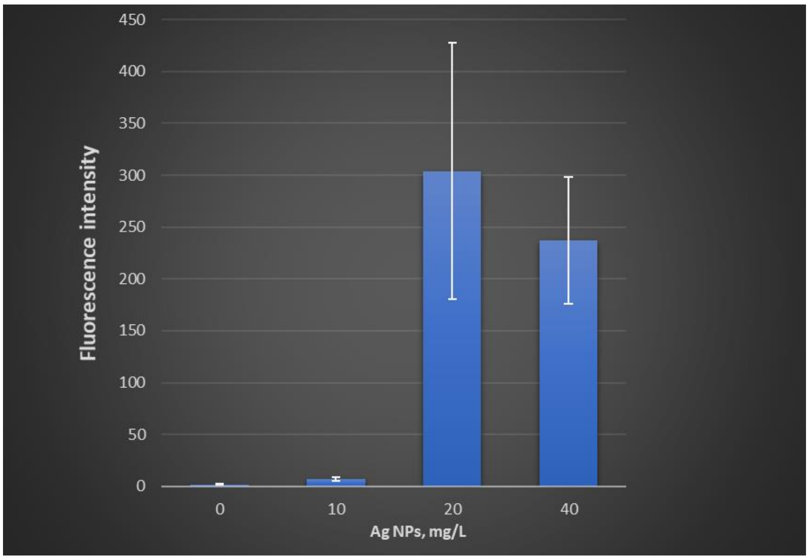

Figure S2. The fluorescence intensity of DCF in root tips of 3-days-old seedlings of wheat cv. ‘Ostka Strzelecka’ developing in Ag NPs suspension at 0, 10, 20 and 40 mg/L.

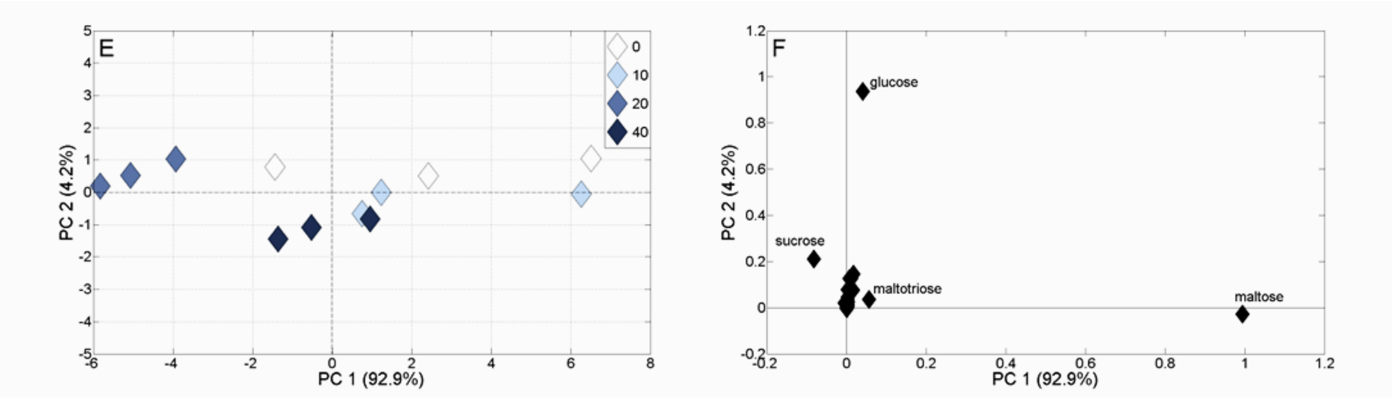

Figure S3. PCA of metabolic profiles of the endosperm of 3-days-old seedlings of wheat cv. ‘Ostka Strzelecka’ developing in Ag NPs suspension at 0, 10, 20 and 40 mg/L.
