# Supplementary material for: The Effect of Bio-Synthesized Silver Nanoparticles on Germination, Early Seedling Development, and Metabolome of Wheat (Triticum aestivum L.)
Source: Molecules. 2022 Apr 1;27(7):2303. doi: 10.3390/molecules27072303 (PMC9000288; doi:10.3390/molecules27072303)
Supplement: Supplementary file 1 [file molecules-27-02303-s001.zip › Supplementary_Tables_S1-S4.pdf]

Table S1. The parameters of polar metabolites identification in GC-MS analyses. RT – retention time, RRT – relative retention time, RI – retention index, ID – type of identification of each metabolite – using mass spectra of original standards (S) or from NIST 05 library only (L), P – the percentage of similarity of identified metabolite to the standard or metabolite from the library.

Metabolites	RT	RRT	RI	ID	P
Soluble carbohydrate					
fructose	13.942, 14.200	1.168, 1.190	1917, 1935	S, L	94
galactose	14.220	1.192	1940	S, L	95
glucose	14.350, 14.592	1.203, 1.223	1946, 1963	S, L	96
sucrose	23.350	1.957	2719	S, L	98
maltose	24.200, 24.457	2.028, 2.050	2827, 2528	S, L	93
maltotriose	30.862, 31.300	2.586, 2.623	3637, 3693	S	95
1-kestose	30.088	2.521	3522	S	95
myo-inositol	17.275	1.448	2142	S, L	95
Amino acids					
alanine	4.183	0.351	908	S, L	98
asparagine	9.992, 11.050	0.837, 0.926	1414, 1494	S, L	97
aspartic acid	7.675, 9.008	0.643, 0.755	1235, 1339	L	96
GABA	9.133	0.765	1348	L	94
glutamic acid	10.325	0.865	14,39	S, L	98
glutamine	12.375	1.037	1696	S, L	89
glycine	6.292	0.527	1121	S, L	93
hydroxyproline	9.050	0.758	1342	L	96
isoleucine	6.117	0.513	1106	S, L	89
leucine	4.658	0.390	962	S, L	94
methionine	7.558	0.633	1226	L	94
phenylalnine	9.350, 10.458	0.784, 0.876	1365, 1449	S, L	96
proline	4.875, 6.183	0.409, 0.518	987, 1112	S, L	93
serine	5.683, 6.908	0.476, 0.579	1066, 1173	S, L	96
treonine	7.267	0.609	1203	L	93
tryptophan	18.817	1.577	2257	L	95
valine	4.038, 5.258	0.338, 0.441	897, 1025	S, L	96
Organic acids					
citric acid	13.092	1.097	1847	S, L	90
fumaric acid	6.650	0.557	1152	S, L	89
lactic acid	3.850	0.323	871	S, L	91
malic acid	8.575	0.719	1306	S, L	96
oxalic acid	4.525	0.379	947	S, L	85
propionic acid	6.542	0.548	1142	L	91
Other					
phosphoric acid	5.900	0.494	1087	S, L	92
urea	5.458	0.457	1044	L	92

Table S2. The concentration of total identified polar metabolites (TIPMs), including total soluble carbohydrates (TSCs), total amino acids (TPAAs), total organic acids (TOAs), and total remaining compounds (TRCs) in roots of 3-day-old seedlings of wheat (*Triticum aestivum* L., cv. 'Ostka Strzelecka') developing in presence of (Bio)Ag NPs at different concentrations. Means of 3 replicates. The same letters by the values indicate no statistically significant differences ($P<0.05$) based on ANOVA analysis and Tukey's post- hoc corrections.

	(Bio)Ag NPs concentration			
	0 mg/L	10 mg/L	20 mg/L	40 mg/L
Metabolites	mg/g^{-}DW			
TIPMs, including:	60.41^{b}	59.47^{b}	75.25^{a}	78.62^{a}
TSCs, including:	38.49^{b}	35.30^{b}	50.63^{a}	56.17^{a}
fructose	9.89^{a}	6.30^{a}	7.69^{a}	7.23^{a}
galactose	5.49^{a}	4.37^{ab}	3.81^{b}	1.54^{c}
glucose	18.90^{a}	11.99^{b}	11.54^{b}	10.16^{b}
1-kestose	0.11^{c}	0.30^{c}	1.21^{b}	1.71^{a}
maltose	0.71^{c}	0.98^{bc}	1.41^{ab}	1.67^{a}
myo-inositol	0.50^{a}	0.39^{c}	0.44^{bc}	0.46^{ab}
sucrose	2.89^{d}	10.98^{c}	24.52^{b}	33.40^{a}
TAAs, including:	9.79^{a}	10.06^{a}	11.41^{a}	11.69^{a}
alanine	0.22^{a}	0.24^{a}	0.19^{a}	0.21^{a}
asparagine	0.10^{c}	1.29^{b}	1.76^{ab}	2.13^{a}
aspartic acid	1.20^{b}	1.79^{a}	1.89^{a}	1.93^{a}
GABA	0.32^{a}	0.23^{a}	0.34^{a}	0.34^{a}
glutamic acid	2.43^{a}	0.04^{b}	0.08^{b}	0.10^{b}
glutamine	0.64^{a}	0.39^{a}	0.72^{a}	0.77^{a}
glycine	0.27^{c}	0.30^{bc}	0.38^{a}	0.36^{ab}
hydroxyproline	1.30^{b}	1.90^{ab}	1.96^{ab}	1.98^{a}
isoleucine	0.33^{a}	0.43^{a}	0.41^{a}	0.43^{a}
leucine	0.48^{a}	0.81^{a}	0.76^{a}	0.57^{a}
methionine	0.04^{a}	0.04^{a}	0.04^{a}	0.03^{a}
phenylalanine	0.43^{a}	0.12^{b}	0.18^{ab}	0.18^{ab}
proline	0.67^{b}	1.07^{a}	1.22^{a}	1.11^{a}
serine	0.37^{b}	0.44^{b}	0.53^{a}	0.57^{a}
threonine	0.12^{a}	0.09^{a}	0.07^{a}	0.10^{a}
tryptophan	0.44^{a}	0.14^{b}	0.16^{b}	0.14^{b}
valine	0.41^{b}	0.75^{a}	0.72^{a}	0.71^{a}
TOAs, including:	8.11^{a}	8.52^{a}	7.43^{a}	5.23^{b}
citric acid	0.16^{c}	3.04^{a}	3.06^{a}	2.22^{b}
fumaric acid	0.03^{a}	0.04^{a}	0.05^{a}	0.05^{a}
lactic acid	1.25^{a}	0.10^{bc}	0.15^{b}	0.07^{c}
malic acid	6.51^{a}	5.22^{b}	4.05^{c}	2.74^{d}
oxalic acid	0.14^{a}	0.09^{b}	0.10^{ab}	0.11^{ab}
propionic acid	0.03^{a}	0.03^{a}	0.04^{a}	0.04^{a}
TRCs, including:	4.02^{b}	5.58^{a}	5.78^{a}	5.54^{a}
phosphoric acid	4.01^{b}	5.58^{a}	5.78^{a}	5.54^{a}
urea	0.00^{a}	0.00^{b}	0.00^{b}	0.00^{b}

Table S3. The concentration of total identified polar metabolites (TIPMs), including total soluble carbohydrates (TSCs), total amino acids (TPAAs), total organic acids (TOAs), and total remaining compounds (TRCs) in coleoptile of 3-day-old seedlings of wheat (*Triticum aestivum* L., cv. 'Ostka Strzelecka') developing in presence of (Bio)Ag NPs at different concentrations. Means of 3 replicates. The same letters by the values indicate no statistically significant differences ($P<0.05$) based on ANOVA analysis and Tukey's post- hoc corrections.

	(Bio)Ag NPs concentration			
	0 mg/L	10 mg/L	20 mg/L	40 mg/L
Metabolites	mg/g DW			
TIPMs, including:	149.63^{a}	96.57^{b}	93.91^{b}	91.21^{b}
TSCs, including:	115.58^{a}	66.70^{b}	68.84^{b}	65.66^{b}
fructose	45.04^{a}	19.12^{b}	22.43^{b}	18.49^{b}
galactose	8.17^{b}	13.79^{a}	10.51^{ab}	13.45^{ab}
glucose	53.01^{a}	23.59^{b}	27.06^{b}	24.76^{b}
1-kestose	3.21^{ab}	3.67^{a}	2.95^{ab}	2.48^{b}
maltose	0.52^{a}	0.31^{a}	0.39^{a}	0.44^{a}
myo-inositol	1.99^{ab}	1.97^{ab}	1.78^{b}	2.05^{a}
sucrose	3.64^{a}	4.25^{a}	3.71^{a}	3.98^{a}
TAAs, including:	9.12^{a}	7.11^{b}	5.85^{bc}	5.29^{c}
alanine	0.21^{ab}	0.23^{a}	0.18^{ab}	0.10^{b}
asparagine	1.95^{a}	1.65^{a}	1.47^{ab}	0.89^{b}
aspartic acid	0.80^{a}	0.52^{b}	0.39^{b}	0.78^{a}
GABA	1.55^{a}	1.11^{b}	0.91^{b}	0.89^{b}
glutamic acid	0.15^{ab}	0.19^{a}	0.17^{ab}	0.09^{b}
glutamine	0.22^{a}	0.21^{a}	0.18^{ab}	0.16^{b}
glycine	0.79^{a}	0.63^{b}	0.45^{c}	0.47^{c}
hydroxyproline	1.16^{a}	1.08^{a}	0.83^{b}	0.62^{c}
isoleucine	0.21^{a}	0.15^{b}	0.12^{c}	0.13^{bc}
leucine	0.20^{a}	0.14^{b}	0.12^{b}	0.14^{b}
methionine	0.03^{a}	0.03^{ab}	0.02^{bc}	0.02^{c}
phenylalanine	0.14^{a}	0.07^{b}	0.06^{b}	0.05^{b}
proline	0.46^{a}	0.29^{b}	0.27^{b}	0.33^{b}
serine	0.55^{a}	0.42^{b}	0.37^{b}	0.36^{b}
threonine	0.06^{a}	0.06^{a}	0.05^{a}	0.01^{b}
tryptophan	0.31^{a}	0.08^{b}	0.05^{b}	0.04^{b}
valine	0.34^{a}	0.25^{b}	0.23^{b}	0.21^{b}
TOAs, including:	16.06^{a}	14.39^{ab}	12.25^{b}	12.57^{b}
citric acid	12.27^{a}	11.11^{a}	9.52^{a}	10.09^{a}
fumaric acid	0.64^{a}	0.63^{a}	0.46^{b}	0.31^{c}
lactic acid	0.26^{a}	0.06^{b}	0.07^{b}	0.06^{b}
malic acid	2.56^{a}	2.26^{b}	1.96^{c}	1.82^{c}
oxalic acid	0.16^{a}	0.14^{a}	0.11^{b}	0.16^{a}
propionic acid	0.17^{a}	0.18^{a}	0.13^{b}	0.13^{b}
TRCs, including:	8.88^{a}	8.37^{ab}	6.98^{c}	7.70^{bc}
phosphoric acid	8.85^{a}	8.35^{ab}	6.96^{c}	7.68^{bc}
urea	0.02^{ab}	0.02^{a}	0.02^{c}	0.02^{bc}

Table S4. The concentration of total identified polar metabolites (TIPMs), including total soluble carbohydrates (TSCs), total amino acids (TPAAs), total organic acids (TOAs), and total remaining compounds (TRCs) in endosperm of 3-day-old seedlings of wheat (*Triticum aestivum* L., cv. 'Ostka Strzelecka') developing in presence of (Bio)Ag NPs at different concentrations. Means of 3 replicates. The same letters by the values indicate no statistically significant differences ($P<0.05$) based on ANOVA analysis and Tukey's post-hoc corrections.

	(Bio)Ag NPs concentration			
	0 mg/L	10 mg/L	20 mg/L	40 mg/L
Metabolites	mg/g DW			
TIPMs, including:	65.30^{a}	63.59^{a}	57.06^{a}	58.82^{a}
TSCs, including:	60.24^{a}	59.28^{a}	52.63^{a}	55.25^{a}
fructose	0.72^{a}	0.51^{b}	0.45^{c}	0.30^{d}
galactose	0.31^{a}	0.24^{ab}	0.09^{b}	0.06^{b}
glucose	9.94^{a}	8.94^{b}	9.38^{ab}	7.93^{c}
1-kestose	2.00^{a}	2.02^{a}	2.12^{a}	2.11^{a}
maltose	34.22^{a}	34.54^{a}	26.90^{b}	31.56^{ab}
maltotriose	1.93^{a}	1.82^{a}	1.43^{b}	1.67^{ab}
myo-inositol	0.31^{a}	0.28^{a}	0.27^{ab}	0.24^{b}
sucrose	10.80^{a}	10.93^{a}	11.99^{a}	11.38^{a}
TAAs, including:	2.94^{a}	2.59^{a}	2.69^{a}	2.14^{b}
alanine	0.12^{a}	0.12^{a}	0.12^{a}	0.10^{a}
asparagine	0.05^{a}	0.04^{b}	0.04^{b}	0.03^{c}
aspartic acid	0.01^{a}	0.01^{a}	0.01^{ab}	0.01^{b}
GABA	0.14^{a}	0.12^{a}	0.11^{a}	0.11^{a}
glutamic acid	0.15^{a}	0.14^{a}	0.16^{a}	0.14^{a}
glutamine	0.07^{a}	0.05^{b}	0.06^{b}	0.03^{c}
glycine	0.17^{a}	0.14^{ab}	0.14^{bc}	0.11^{c}
hydroxyproline	0.38^{a}	0.36^{a}	0.37^{a}	0.31^{a}
isoleucine	0.13^{a}	0.11^{ab}	0.12^{ab}	0.10^{b}
leucine	0.26^{a}	0.22^{ab}	0.23^{ab}	0.17^{b}
methionine	0.01^{a}	0.01^{a}	0.01^{ab}	0.01^{b}
phenylalanine	0.33^{a}	0.28^{b}	0.30^{ab}	0.22^{c}
proline	0.59^{a}	0.51^{ab}	0.53^{a}	0.42^{b}
serine	0.19^{a}	0.17^{a}	0.17^{a}	0.13^{b}
threonine	0.03^{a}	0.03^{a}	0.03^{a}	0.03^{a}
tryptophan	0.07^{a}	0.04^{a}	0.06^{a}	0.04^{a}
valine	0.24^{a}	0.22^{a}	0.23^{a}	0.18^{b}
TOAs, including:	1.11^{a}	0.81^{b}	0.82^{b}	0.72^{b}
citric acid	0.76^{a}	0.52^{b}	0.51^{b}	0.41^{b}
fumaric acid	0.01^{a}	0.01^{b}	0.01^{bc}	0.01^{c}
lactic acid	0.12^{a}	0.07^{b}	0.08^{b}	0.07^{b}
malic acid	0.11^{a}	0.11^{a}	0.10^{a}	0.11^{a}
oxalic acid	0.10^{a}	0.11^{a}	0.12^{a}	0.12^{a}
propionic acid	0.01^{a}	0.01^{a}	0.01^{a}	0.01^{a}
TRCs, including:	1.01^{a}	0.91^{a}	0.91^{a}	0.72^{b}
phosphoric acid	1.01^{a}	0.91^{a}	0.91^{a}	0.71^{b}
urea	0.01^{a}	0.00^{b}	0.01^{b}	0.01^{b}
